# Overactivity of Alternative Pathway Convertases in Patients With Complement-Mediated Renal Diseases

**DOI:** 10.3389/fimmu.2018.00612

**Published:** 2018-04-04

**Authors:** Marloes A. H. M. Michels, Nicole C. A. J. van de Kar, Marcin Okrój, Anna M. Blom, Sanne A. W. van Kraaij, Elena B. Volokhina, Lambertus P. W. J. van den Heuvel

**Affiliations:** ^1^Department of Pediatric Nephrology, Radboud Institute for Molecular Life Sciences, Amalia Children’s Hospital, Radboud University Medical Center, Nijmegen, Netherlands; ^2^Department of Medical Biotechnology, Intercollegiate Faculty of Biotechnology, University of Gdańsk and Medical University of Gdańsk, Gdańsk, Poland; ^3^Medical Protein Chemistry, Department of Translational Medicine, Lund University, Malmö, Sweden; ^4^Department of Laboratory Medicine, Radboud University Medical Center, Nijmegen, Netherlands; ^5^Department of Pediatrics/Pediatric Nephrology and Department of Development and Regeneration, University Hospitals Leuven, Leuven, Belgium

**Keywords:** complement system, alternative pathway, convertase, C3 nephritic factor, C3 glomerulopathy, atypical hemolytic uremic syndrome, complement factor B mutation

## Abstract

Overactivation of the alternative pathway of the complement system is associated with the renal diseases atypical hemolytic uremic syndrome (aHUS) and C3 glomerulopathy (C3G). C3 nephritic factors (C3NeF) play an important role in C3G pathogenesis by stabilizing the key enzymatic complex of complement, the C3 convertase. However, the reliability of assays detecting these autoantibodies is limited. Therefore, in this study, we validated and optimized a prototype hemolytic method for robust detection and characterization of factors causing convertase overactivity in large patient cohorts. The assay assesses convertase activity directly in the physiological milieu of serum and therefore is not restricted to detection of stabilizing autoantibodies such as C3NeF but may also reveal genetic variants resulting in prolonged convertase activity. We first defined clear cutoff values based on convertase activity in healthy controls. Next, we evaluated 27 C3G patient samples and found 16 positive for prolonged convertase activity, indicating the presence of factors influencing convertase stability. In three patients, the overactive convertase profile was persistent over disease course while in another patient the increased stability normalized in remission. In all these four patients, the convertase-stabilizing activity resided in the purified immunoglobulin (Ig) fraction, demonstrating the autoantibody nature. By contrast, the Igs of a familial aHUS patient carrying the complement factor B mutation p.Lys323Glu did not reveal convertase stabilization. However, in serum prolonged convertase activity was observed and segregated with the mutation in both affected and unaffected family members. In conclusion, we present a robust and reliable method for the detection, characterization, and evaluation over time of factors prolonging convertase activity (C3NeF or certain mutations) in patient cohorts. This assay may provide new insights in disease pathogenesis and may contribute to the development of more personalized treatment strategies.

## Introduction

The complement system, a cornerstone of innate immunity, provides the body with protection against invading pathogens and dangerous host cells (1, 2). The system can be activated *via* three pathways—the classical, lectin and alternative pathway (AP)—depending on the initial trigger that is encountered. All pathways converge at the central event of complement activation: the cleavage of C3 by C3 convertases. This enzymatic reaction supports further activation of the complement cascade with the release of various anaphylatoxins and opsonins, and eventually the formation of the membrane attack complex (MAC). These events all contribute to inflammation, phagocytosis and the damage of susceptible targets (1–3).

In contrast to the classical and lectin pathway, which are only activated upon pattern recognition, the AP is continuously mildly activated and therefore specifically serves as a surveillance mechanism. C3 is subjected to spontaneous hydrolysis of its internal thioester bond at a very low rate, and this generates the active C3(H_2_O) molecule. Hydrolyzed C3 can bind to complement factor B (FB), which is subsequently cleaved by complement factor D (FD) to form an initial, fluid phase AP C3 convertase, C3(H_2_O)Bb, that is capable of converting native C3 into the active C3a (anaphylatoxin) and C3b (opsonin) fragments. This mechanism providing a persistent low level of active C3 in the blood is known as “tick-over” and allows constant responsiveness to potential danger. Besides the tick-over mechanism, the AP may also be initiated by C3 convertases of the classical and lectin pathway that generate active C3b (1, 4, 5). Furthermore, C3 may be cleaved into C3b by certain non-specific proteases, especially at sites of inflammation, coagulation, and infection (6).

Activated C3b molecules expose a reactive thioester moiety *via* which they can subsequently bind to hydroxyl or amino groups on target surfaces in close proximity to the activation site. This C3b binding, and thus initiation of AP activity, is preferentially triggered by certain carbohydrate structures on microbes and other foreign surfaces, e.g., LPS and zymosan. By interacting with FB and FD, target-bound C3b can subsequently form new surface-bound convertases (C3bBb) that can be further stabilized by properdin (C3bBbP). Properdin has also been proposed to act as a pattern recognition molecule able to initiate the AP on certain targets by recruiting C3b and FB. Once formed, AP C3 convertases can massively amplify the immune response by converting more C3 molecules into C3b, which in turn support new convertase formation. Incorporation of C3b into existing C3 convertases generates C5 convertases (C3bBbC3b), which cleave C5 into the potent anaphylatoxin C5a and the fragment C5b that initiates the assembly of the MAC (C5b-9) complex. The amplification loop of the AP can also be induced by C3b molecules generated by C3 convertases from the other two activation pathways. In this way, the AP can account for over 80% of total complement activity. Thus, besides being a surveillance system, the AP acts as an important amplifier of initiated complement responses (1, 4, 5).

The powerful action of the AP requires sophisticated regulation to prevent damage by excessive activation or self-attack. Therefore, human cells express membrane-bound complement inhibitors, including decay-accelerating factor (DAF), membrane cofactor protein (MCP), and complement receptor 1 (CR1). Together with the soluble regulatory proteins complement factor H (FH) and complement factor I (FI), which can act in fluid phase or can be recruited to surfaces, these complement regulators prevent the formation of potent C3 convertases on healthy host surfaces and keep AP activity in control. The inhibitors of the C3 convertase can act *via* two ways. The first is by accelerating the convertase decay and is fulfilled by DAF, CR1, and FH. These regulators can also compete with FB for binding to C3b and thereby inhibit new convertase formation. The second is by preventing convertase formation *via* acting as cofactors for FI-mediated inactivation of C3b. This action is supported by MCP, CR1, and FH (7–9). However, genetic aberrations in complement genes and/or autoantibodies against complement components may, in combination with triggering events, disturb this sophisticated regulation, causing overactivation of the system. A dysregulated AP has been particularly associated with the renal diseases the atypical hemolytic uremic syndrome (aHUS) and the disease entity C3 glomerulopathy (C3G) (10–15).

C3 glomerulopathy is a recently defined umbrella classification for severe renal diseases that are characterized by C3 accumulation in the glomeruli without or with sparse immunoglobulin (Ig) deposition. The main two diseases encompassed by C3G are dense deposit disease (DDD) and C3 glomerulonephritis (C3GN) (16). Up to 50% of patients progress to end-stage renal disease within 10 years after first presentation (17). The most important pathogenic factors in C3G are autoantibodies against the C3 convertase of the AP named C3 nephritic factors (C3NeF), although pathogenic variants in complement genes have also been reported (14, 15). By binding to neoepitopes of the formed C3bBb(P), C3NeF stabilizes the otherwise labile convertase and thereby prolongs its half-life (18). C3NeF has been detected in approximately 80–90% of DDD patients (15, 19, 20) and in 40–50% of patients with C3GN (15, 21).

Atypical HUS is a form of thrombotic microangiopathy and is not associated with C3NeF, but pathogenic mutations in complement genes or autoantibodies against FH are the most common cause of improper complement regulation (22–25). Blocking C5 and terminal pathway activation with the C5-directed antibody eculizumab is a very effective treatment for this disease. Eculizumab became the first approved complement-inhibiting drug for aHUS in 2012 (26, 27). By contrast, trials of eculizumab treatment in C3G patients have shown contradicting outcomes so far. Thus, the value of eculizumab as a therapy for C3G requires further investigation (17, 28, 29).

Detection of C3NeF is not only helpful in diagnosis of C3G but also in choice of therapy for the patient, especially in the light of upcoming complement-inhibiting strategies. Nevertheless, a robust and reproducible method for C3NeF detection is still lacking. A recent European quality assessment round reported a success rate of only 50% among the participating laboratories (30). This also hampers our understanding of the exact role of C3NeF in C3G pathogenesis. C3NeF are functionally heterogeneous (31) and conflicting associations have been found between its presence and the patient’s disease state and progression (20, 32–34). Some reports even describe C3NeF in healthy individuals (35, 36).

Recently, we described a new hemolytic method for measurement of convertase activity in the physiological milieu of whole serum. This assay allows the robust detection of factors prolonging convertase stability in a direct way, such as C3NeF, and/or in an indirect way by impairing the regulation of the convertase (37). For convenience, we will use the term convertase-stabilizing factors for both these types of factors that are either directly or indirectly prolonging the convertase half-life. The assay uses C5-blocking agents [eculizumab or the tick protein *Ornithodoros moubata* complement inhibitor (OmCI) (37, 38)] to strictly separate the complement cascade into two steps: the formation of C3/C5 convertases by test sera in a first step and the formation of lytic MAC complexes in a standardized second step for readout. Dynamics of convertase assembly and decay in the first step are then monitored in time. In this work, we further modified this convertase activity assay to screen for convertase-stabilizing factors in patient cohorts by defining clear cutoff criteria based on healthy controls. In addition, we showed expanded analysis of clinical samples with different types of convertase-stabilizing factors and their presence over disease course.

## Materials and Methods

### Sample Collection

Whole blood was obtained from patients and healthy controls and processed within 1 h after sampling. For serum, blood was allowed to clot at room temperature for 30–45 min followed by centrifugation (10 min, 2,500 *g*, 4°C). To obtain plasma, blood was drawn into tubes with the anticoagulant ethylenediaminetetraacetic acid (EDTA) that were immediately placed on ice and subsequently centrifuged according to the same procedure. Afterward, the serum and EDTA plasma fractions were collected, aliquoted, and stored at −80°C until use. For the healthy controls, following exclusion criteria were applicable: fever, bacterial/viral infection in previous 2 weeks, chronic illness, inherited or acquired immune disorders, and immunosuppressive medication. In addition, pooled normal human serum (NHS) and pooled normal human plasma (NHP) were made by pooling 15 and 12 individual control samples, respectively. This study was carried out in accordance with the recommendations of the appropriate version of the Declaration of Helsinki. All subjects gave written informed consent, and the protocol was approved by the ethical committee of the Radboud University Medical Center (FH 06979).

### Erythrocyte Working Suspensions

Rabbit erythrocytes (RbE) in Alsever’s solution were obtained from Envigo (Venray, the Netherlands). The working suspensions of RbE were prepared by washing them with magnesium–ethylene glycol tetraacetic acid (Mg–EGTA) buffer (2.03 mM veronal buffer, pH 7.4, 10 mM EGTA, 7 mM MgCl_2_, 0.083% gelatin, 115 mM d-glucose, and 60 mM NaCl) until there was no visible hemoglobin left in the supernatant. To standardize the number of erythrocytes in each experiment, these working suspensions were calibrated so the absorbance measured at 405 nm of 10× diluted erythrocytes in water was between 0.8 and 1.2.

### Ig Purification

The Ig fractions from NHP and from the EDTA plasma of patients P24, P25, P26, and P27 (Table 1) were isolated by affinity purification using NAb™ protein A/G 5 mL spin columns (Thermo Fisher Scientific, Waltham, MA, USA) according to the manufacturer’s protocol. In short, 0.85–1.0 mL of EDTA plasma was diluted with binding buffer (0.1 M phosphate, 0.15 M sodium chloride, and pH 7.2) to 10 mL and loaded on the columns. Unbound fractions were washed with binding buffer, and thereafter bound Igs were eluted using 0.1 M glycine, pH 2.5 followed by immediate neutralization with 1/10 volume 1 M Tris, pH 8.5. Subsequently, Ig fractions were dialyzed against phosphate buffered saline using SnakeSkin^®^ dialysis tubes (Thermo Fisher Scientific, Waltham, MA, USA) with a 10 kD molecular weight cutoff and concentrated to the initial plasma sample volume using Amicon^®^ Ultra-15 centrifugal filter devices (Merck Millipore, Billerica, MA, USA) with similar cutoff. Total protein concentrations in these samples were measured using NanoDrop Spectrophotometer (Thermo Fisher Scientific, Waltham, MA, USA) and varied between 4.73 and 12.48 mg/mL, which are within the normal range for Igs.

**Table 1:** Clinical and genetic data.

Patient/subject	Gender (M/F)	Age at time of study (years)	Diagnosis	Genetic aberrations^b^	Convertase-stabilizing factors present (Y/N)	C3 levels^g^ (mg/L; 700–1500)
**(a) C3G cohort**						
P1	M	63	DDD		N	NA
P2	M	10	C3GN		N	NA
P3	M	66	C3GN		N	588
P4	M	52	DDD	*C3* c.26T>C (p.Leu9Pro)^c^ located in signal sequence	N	NA
P5	M	43	C3GN		N	Normal
P6	F	42	C3GN		N	Normal
P7	M	37	C3GN		N	941
P8	F	33	C3G^a^		N	NA
P9	M	6	DDD		N	644
P10	M	9	C3G^a^		N	660
P11	M	11	C3GN		N	530
P12	F	44	C3GN		Y	102
P13	M	7	DDD	*CFI* c.1217G>A (p.Arg406His)^d^ (39)	Y	Decreased
P14	F	19	C3G^a^	*CFH* c.2850G>T (p.Gln950His)^e^ (15), ^f^(40)	Y	NA
P15	M	27	C3GN	*C3* c.962G>A (p.Gly321Glu)^c^	Y	NA
P16	F	6	C3GN	*CFH* full length deletion^c^	Y	500
P17	F	7	C3G^a^		Y	NA
P18	M	10	C3GN		Y	220
P19	M	7	DDD		Y	210
P20	F	9	DDD		Y	100
P21	F	7	DDD		Y	510
P22	M	5	DDD		Y	110
P23	M	15	C3GN		Y	NA
P24	F	8	C3GN	*CFHR5* c.542G>C (p.Arg181Thr)^c^	Y	110
P25	M	9	DDD		Y	82
P26	F	5	C3GN		Y	830
P27	M	15	C3GN		Y	70
**(b) Family with complement factor B mutation and aHUS**						
II. 5	M	41	–		N	
II. 6	F	40	–	*CFB* c.967A>G (p.Lys323Glu)^f^ (41, 42)	Y	
III. 1	F	22	–		N	
III. 2	M	18	–		N	
III. 4	F	14	aHUS	*CFB* c.967A>G (p.Lys323Glu)^f^ (41, 42)	Y	
III. 5	F	7	–		N	
III. 6	F	4	aHUS	*CFB* c.967A>G (p.Lys323Glu)^f^ (41, 42)	Y	260

*^a^No distinction possible into C3GN or DDD, since no electron microscopy was performed or no data available*.

*^b^All genetic variants found were heterozygous*.

*^c^To the best of our knowledge, not previously reported in context of C3G*.

*^d^Previously reported in a DDD patient*.

*^e^Previously reported in C3GN patients*.

*^f^Previously reported in aHUS patients*.

*^g^C3 levels were measured by nephelometry with the normal range indicated between brackets. Data given are those available as closest to the time of convertase activity assessment. For samples obtained from peripheral centers and from which C3 data were accessible, results are presented as “normal” or “decreased” since normal ranges vary between centers*.

*aHUS, atypical hemolytic uremic syndrome; C3G, C3 glomerulopathy; C3GN, C3 glomerulonephritis; DDD, dense deposit disease; NA, not available*.

### Convertase Activity Assay

**Figure 1:**
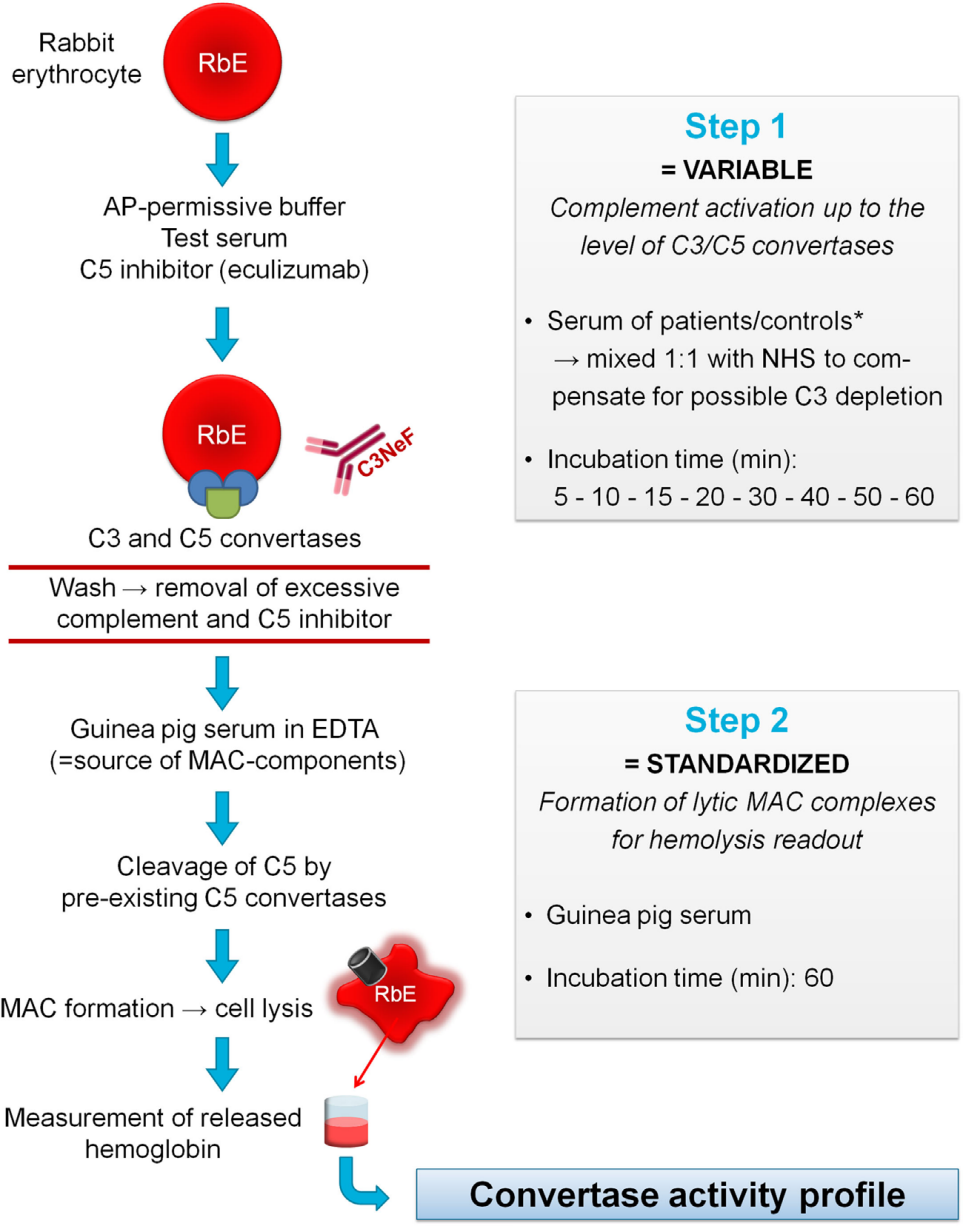
Principles of the convertase activity assay for detecting convertase-stabilizing factors in serum. In step 1, rabbit erythrocytes (RbE) are incubated in an alternative pathway (AP)-permissive buffer with test serum mixed 1:1 with pooled normal human serum (NHS) to compensate for possible C3 depletion in the test sample. The C5 inhibitor eculizumab is added to halt complement activation at the level of the C3/C5 convertases. Convertase assembly and decay are followed over time using different incubation times (5–60 min). Convertase-stabilizing factors present in the sample, e.g., C3 nephritic factor (C3NeF), may interfere at this point with convertase decay. Before proceeding to step 2, erythrocytes are washed to remove remaining complement factors and C5 inhibitor. Then, convertase-bearing erythrocytes are incubated for 60 min with guinea pig serum as a source of membrane attack complex (MAC) components. The presence of ethylenediaminetetraacetic acid (EDTA) disables *de novo* formation of convertases from guinea pig serum and assures that only preformed convertases of the first step may initiate MAC formation and subsequent hemolysis. The released hemoglobin is quantified by spectrophotometric measurement and reflects the activity of the preformed convertases in step 1 per experimental time point. These data are used to generate convertase activity profiles over time. *If desired, immunoglobulin fractions may be added to NHS to dissect the nature of the stabilizing factor (see Materials and Methods).

Detection of AP convertase-stabilizing factors in human serum was achieved using a two-step hemolytic assay as previously described (37) and as further specified in Figure 1. Briefly, per experimental time point 10 µL of prepared RbE were mixed with 20 µL Mg–EGTA containing 150 nM of the C5 inhibitor eculizumab (Alexion Pharmaceuticals, Cheshire, CT, USA), after which at different time points an additional 20 µL Mg–EGTA containing test serum or Ig fractions mixed 1:1 with NHS was added for convertase assembly (5, 10, 15, 20, 30, 40, 50, or 60 min). Final concentrations of serum were optimized per batch of erythrocytes and were generally 3.75 or 5%. Subsequently, cells were washed with 150 µL 40 mM EDTA–gelatin veronal buffer (EDTA–GVB, 4.41 mM veronal buffer, 0.1% gelatin, 130 mM NaCl, pH 7.4) and collected by centrifugation (2 min, 1,000 *g*, room temperature). Thereafter, cells were incubated for 60 min with 50 µL of 2.5% guinea pig serum (Envigo, Venray, the Netherlands) in EDTA–GVB and another 50 µL of Mg–EGTA buffer to develop MAC and subsequent hemolysis. All incubation steps were performed at 37°C, with 600 rpm agitation in a VWR^®^ Incubating Microplate Shaker with 3 mm orbit (VWR International, Radnor, PA, USA) in V-shaped 96-well plates (Greiner Bio-One, Kremsmünster, Austria). Finally, supernatant was collected by centrifugation and transferred to flat-bottom 96-well plates (Greiner Bio-One, Kremsmünster, Austria). Hemolysis was quantified as percentage of full lysis by an equal amount of erythrocytes in water: (*A*_405_ test sample − *A*_405_ blank)/(*A*_405_ full lysis − *A*_405_ blank) × 100.

### Statistical Analysis

Where indicated, data were analyzed using two-way analysis of variance with Bonferroni’s post test using GraphPad Prism 5.03 for Windows (GraphPad Software, San Diego, CA, USA).

## Results

### Study Cohort

A group of 27 patients diagnosed with C3G and referred to the Radboud University Medical Center were included in the study. Diagnosis was suspected based on presence of clinical features such as hypertension, proteinuria, and nephrotic/nephritic syndrome, possibly combined with low serum C3. Diagnosis was confirmed after pathological judgment of renal biopsy following the recommendations defined in the consensus report of the first C3G Meeting (16). Subdivision of C3G into C3GN and DDD was solely based on electron microscopy appearance. In total, 9 patients were diagnosed with DDD, 14 with C3GN, and in 4 patients no distinction between the C3G subforms could be made since data required for subdivision were not available. In this C3G cohort (age range: 5–66 years; 17 children, 10 adults), no FH autoantibodies (FHaAb) were detected, and 6 patients carried mutations in complement (regulating) genes (Table 1a). Besides samples from C3G patients, sera from a female patient with familial aHUS and six of her affected and non-affected family members were available for analysis. In total, three of these family members were carrier of the heterozygous FB p.Lys323Glu mutation and two of them were also affected with aHUS (Table 1b).

### Convertase Activity in Healthy Donors

**Figure 2:**
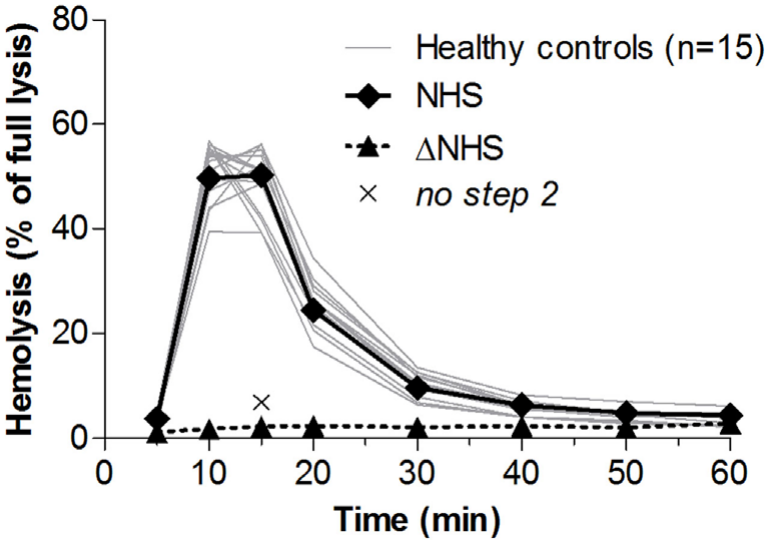
Convertase activity in serum samples of healthy controls. Samples collected from 15 healthy controls were mixed 1:1 with pooled normal human serum (NHS) to an end concentration of 3.75%. Heat-inactivated NHS (ΔNHS) and an NHS sample from which guinea pig serum was omitted during the second part of the assay (*no step 2*) served as negative controls for the first and second steps, respectively. Means are given of ≥2 independent experiments for each control. Error bars were omitted for better visibility of the graph. Hemolysis levels are presented as percentage of full lysis of erythrocytes in water.

To define normal convertase activity, we tested the sera of 15 healthy controls (Figure 2). Due to low serum C3 levels *in vivo*, many C3G samples show low hemolytic activity. To compensate for this possible complement consumption, all samples in this study, including those from controls, were diluted 1:1 with NHS. We previously showed that this approach restored hemolytic activity while still allowing the detection of convertase-stabilizing factors in patient serum (37) (also see Figure S1 in Supplementary Material). The convertase activity profiles of healthy controls demonstrated a constant pattern, which was comparable to that of NHS. Convertase activity reached its maximum after 10–15 min of incubation, and at 30 min activity returned to background levels in all control samples.

The ratio between the highest achieved hemolysis (top) and the lysis at the point at which controls have returned to background levels, in this case at *t* = 30 min (*t*_30_), was 5.4 ± 2.7 (mean ± 2 SD) for healthy controls. Prolonged convertase activity, i.e., delayed decay, was defined as a top/*t*_30_ ratio below the mean − 2 SD of controls, here <2.7. Because samples with a low hemolytic activity overall are also likely to be positive for this criterion, only samples that have a total area under the curve equal to or higher than that of NHS are considered positive.

### Convertase Activity in Patients

**Figure 3:**
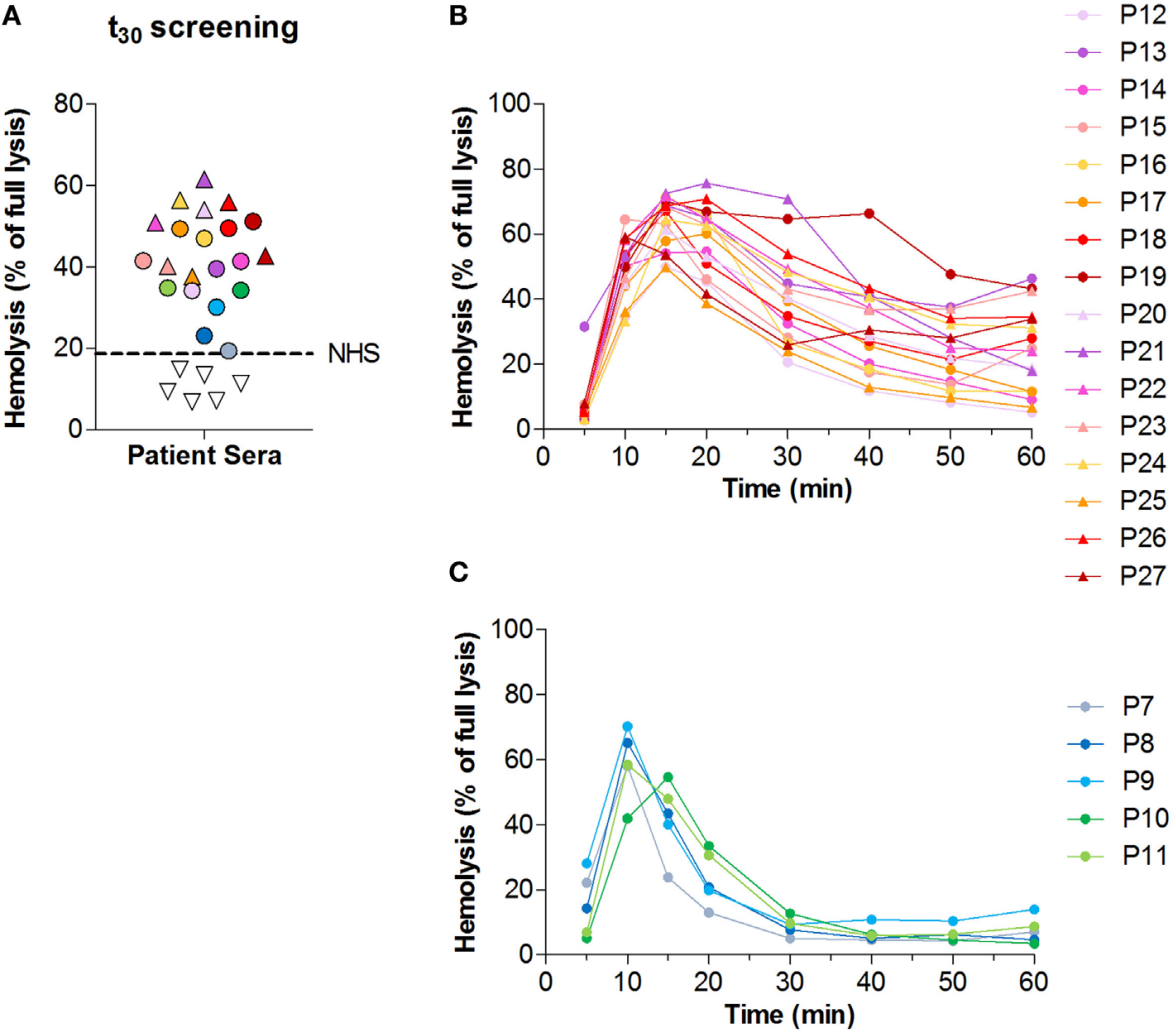
Screening for convertase-stabilizing factors in patients with C3 glomerulopathy (C3G). **(A)** Convertase activity of 27 patients with C3G as measured in a single time point screening with 30 min of incubation time. Samples showing hemolysis above that of pooled normal human serum (NHS), represented by the dotted line, are indicated with colored symbols and were selected for further analysis of convertase activity over time. **(B,C)** Convertase activity profiles of the 21 patients selected from the *t*_30_ screening in panel **(A)**. Samples positive for convertase-stabilizing factors (top/*t*_30_ ratio < 2.7) are given in panel **(B)**; samples negative for the presence of convertase-stabilizing factors (top/*t*_30_ ratio > 2.7) in panel **(C)**. Results given are from a single assay set representative for at least two performed on each sample. **(A–C)** Serum samples were all tested mixed 1:1 with NHS to a final concentration of 3.75%. Colors and symbols correspond to the same patients in all panels. Hemolysis levels are given as percentage of full lysis of erythrocytes in water.

Next, we analyzed convertase activity in the cohort of 27 C3G patients. Samples were first screened at *t*_30_ in a single time point screening (Figure 3A). The 21 samples that at *t*_30_ showed lysis above that of NHS (18.7%) were selected for further analysis of convertase activity over time. All other samples were considered negative for the presence of convertase-stabilizing factors.

Of the 21 patients who were selected for analysis of the convertase activity profile, 16 showed a top/*t*_30_ ratio below 2.7 (Figure 3B). These samples were considered positive for convertase-stabilizing factors. The other five samples, selected in the initial screening as possibly positive, did not meet these criteria and were therefore considered to have normal convertase activity profiles (Figure 3C).

### Convertase Activity Over Disease Course

**Figure 4:**
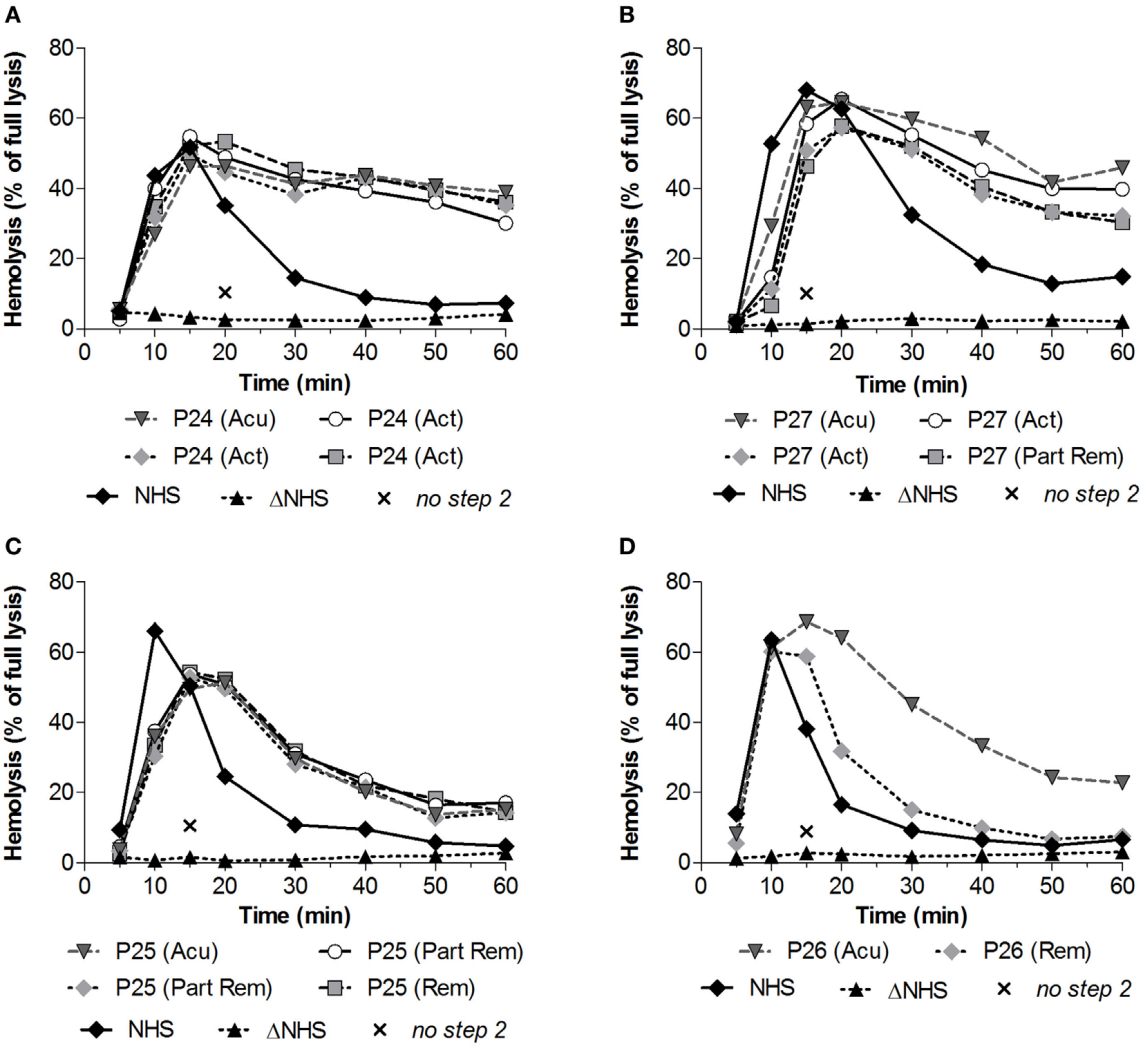
Convertase activity profiles of patients with C3 glomerulopathy over disease course. Patient serum samples of P24 **(A)**, P27 **(B)**, P25 **(C)**, and P26 **(D)** from different collection dates associated with different disease states were tested mixed 1:1 with pooled normal human serum (NHS) to a final concentration of 3.75% in experiments shown in panels **(C,D)** or 5% in the experiments shown in panels **(A,B)**, since they were performed using different batches of erythrocytes. Abbreviations: Acu, acute phase at first clinical presentation of disease; Act, active disease; Part Rem, partial remission; Rem, remission. Heat-inactivated NHS (ΔNHS) and an NHS sample from which guinea pig serum were omitted during the second part of the assay (*no step 2*) served as negative controls for the first and second steps, respectively. Representative data are given. Hemolysis levels are given as percentage of full lysis of erythrocytes in water.

Subsequently, we investigated whether convertase activity profiles could change over disease course. From four patients who showed increased convertase stability, P24–P27 (Table 1), serum samples were available derived over different periods of their disease, e.g., from active disease states and/or (partial) remission periods. The active disease state samples of P24 and the active disease and partial remission samples of P27 all displayed prolonged convertase activity (Figures 4A,B). Also P25 showed a persistent stabilized convertase profile in both active disease and in partial and complete remission (Figure 4C). By contrast, the prolonged convertase activity shown for P26 was only detected in the acute phase and normalized in remission (Figure 4D).

### Prolonged Convertase Activity due to Autoantibodies

**Figure 5:**
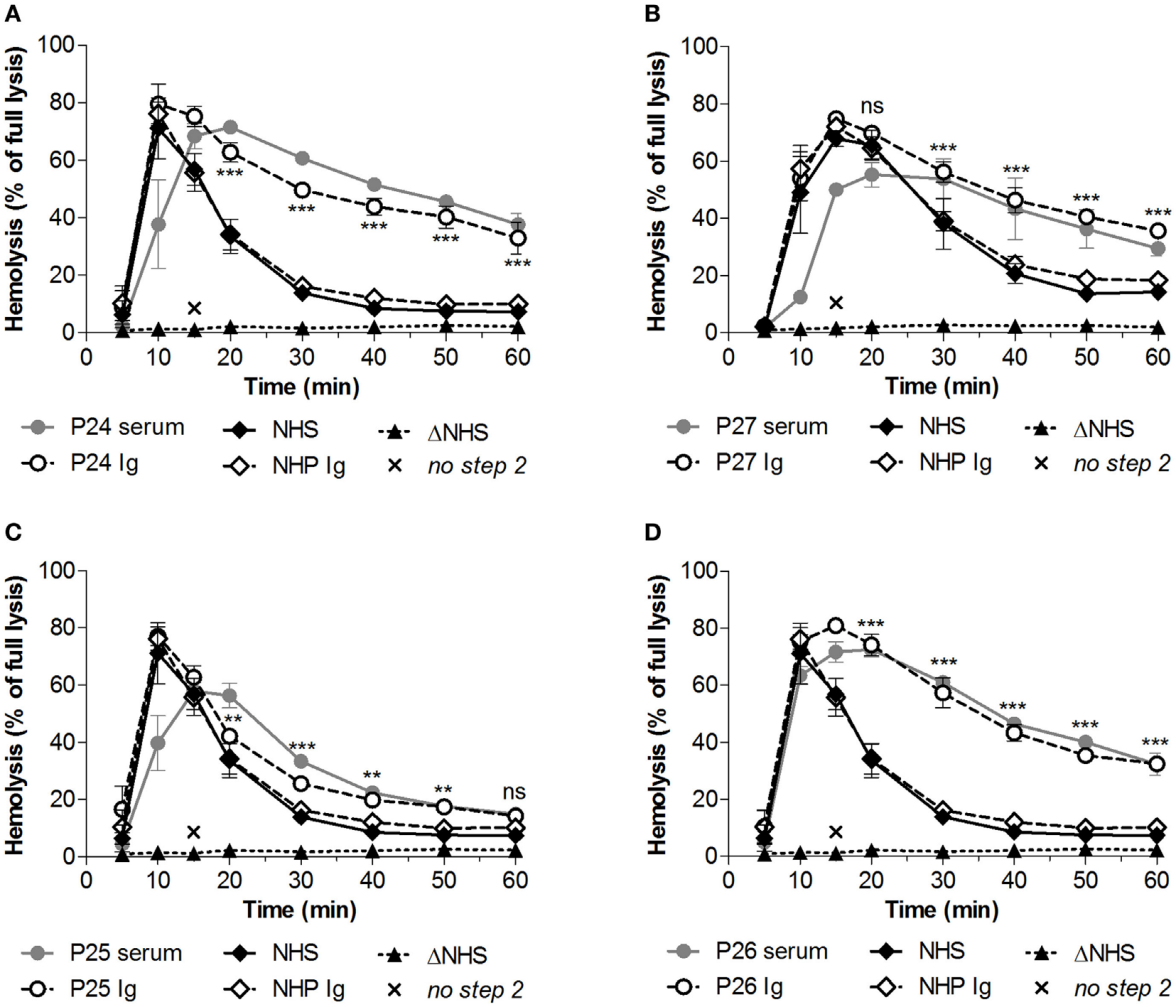
Identification of convertase-stabilizing factors in immunoglobulin (Ig) fractions of patients with C3 glomerulopathy. Serum samples of P24 **(A)**, P27 **(B)**, P25 **(C)**, and P26 **(D)** were tested mixed 1:1 with pooled normal human serum (NHS) to a final concentration of 5%. Alternatively, the Ig fractions of these patients or of pooled normal human plasma (NHP) were added to 5% NHS in an equal volume. Data were collected from three independent experiments; means are given with error bars showing SDs. Statistical analysis for test samples compared with NHP Ig from *t* = 20 to *t* = 60 as calculated using two-way analysis of variance is given for the patient Igs only: **P < 0.01, ***P < 0.001, ns not significant. Heat-inactivated NHS (ΔNHS) and an NHS sample from which guinea pig serum were omitted during the second part of the assay (*no step 2*) served as negative controls for the first and second steps, respectively. Hemolysis levels are given as percentage of full lysis of erythrocytes in water.

For these four patients, we then investigated whether the convertase-stabilizing effect was due to autoantibodies such as C3NeF (Figure 5). To this end, the Ig fractions from these patients were purified using protein A/G affinity chromatography. Samples were concentrated in phosphate buffered saline to the initial plasma sample volume. Addition of an equal volume of the purified Ig fractions to NHS revealed comparable stabilizing effects on the convertases as seen for these patient’s serum samples. This stabilization could not be observed when healthy control Igs were added to NHS. Thus, in these patients prolonged convertase activity could be attributed to autoantibody activity.

### Prolonged Convertase Activity due to FB Mutation in aHUS Family

**Figure 6:**
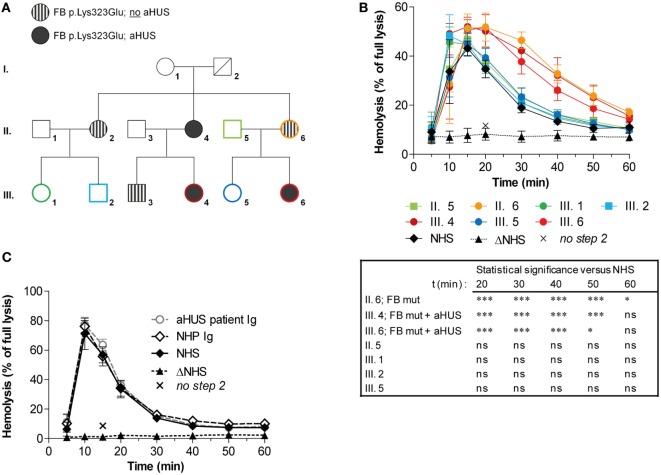
Assessment of convertase activity in a family with complement factor B (FB) mutation and atypical hemolytic uremic syndrome (aHUS). **(A)** Pedigree with carriers of the FB p.Lys323Glu mutation and/or aHUS. No genetic information of this variation was available for I. 2 who is deceased. **(B)** Analysis of convertase stability in available samples from seven family members. The sera were tested mixed 1:1 with normal human serum (NHS) to a final concentration of 5%. Data for each test sample were obtained from three independent experiments; means are given with error bars showing SDs. Statistical analysis for test sera compared with NHS from *t* = 20 to *t* = 60 as calculated using two-way analysis of variance is given: *P < 0.05, ***P < 0.001, ns not significant. **(C)** Assessment of the presence of convertase-stabilizing factors in the immunoglobulin (Ig) fraction of aHUS patient III. 6. Purified Igs from patient plasma or pooled normal human plasma (NHP) were added in an equal volume to 5% NHS. Data were collected from three independent experiments; means are given with error bars showing SDs. **(B,C)** Heat-inactivated NHS (ΔNHS) and an NHS sample from which guinea pig serum were omitted during the second part of the assay (*no step 2*) served as negative controls for the first and second steps, respectively. Hemolysis levels are given as percentage of full lysis of erythrocytes in water.

Previously, we described prolonged convertase activity in the serum of an aHUS patient carrying the p.Lys323Glu mutation in FB (37). In this study, we analyzed convertase activity in available serum samples of affected and unaffected family members of this patient (Figure 6A). Stabilized convertase profiles segregated with the FB mutation in both affected and non-affected family members (Figure 6B). When purified total Igs of the index patient were added to NHS, no stabilization of the C3 convertase could be observed (Figure 6C), unlike Ig fractions from C3G patients (Figure 5). This indicates that in these individuals, prolonged convertase activity could be attributed to the FB mutation rather than to autoantibody activity.

## Discussion

Previously, we described proof-of-concept of a robust method to measure C3 convertase activity allowing detection of factors influencing convertase activity in whole serum. Here, we further standardized assay conditions for screening in patient cohorts and for critical evaluation of convertase-stabilizing factors in clinical samples.

We showed low variation among healthy controls in the assay (Figure 2) and based on this defined clear cutoff criteria for assessing the presence of convertase-stabilizing factors in patient samples. The samples were considered positive when they showed a top/*t*_30_ ratio lower than the mean − 2 SD of healthy controls in their convertase activity profile. Cutoff values have to be evaluated for each laboratory individually. When large cohorts of patients have to be analyzed, the samples may be first screened for high lysis at a single time point (e.g., *t*_30_) and compared with NHS (Figure 3A). This efficient approach allows to save both time and patient material, but we underline that it should supplement and not replace the evaluation of a complete convertase activity profile to confirm positivity. In this work, we only used serum samples to assess convertase activity, but from our unpublished data we know that EDTA plasma samples may be used as well; no complement-inhibiting effects of EDTA are observed under the described test conditions (Figure S2 in Supplementary Material). Furthermore, as an alternative for eculizumab, which is expensive and may be difficult to access, the tick protein OmCI [or its commercially available variant Coversin (43)] may be used for C5 blockage in the first step of the assay (37, 38). Future research may benefit from cheaper alternative C5-blocking agents.

The positive samples showed high variation in the degree of convertase stabilization: where some sera showed active convertases up to *t*_60_, other sera showed delayed decay with breakdown of convertases between 30 and 60 min (Figure 3B). First of all, this may be attributed to the heterogeneity of C3NeF autoantibodies, which recognize different neoepitopes of the convertase complex. All C3NeF prevent the natural intrinsic decay of the convertase (44), but they vary in the degree of resistance they provide against extrinsic decay mediated by complement regulators (31, 44–47). Two reports described C3NeF that were completely unable to interfere with complement regulators and the authors indicated them as likely less pathogenic *in vivo*, since patients with these C3NeF did not present with hypocomplementemia (31, 47). Besides, the interaction of C3NeF with the convertase can be dependent on properdin or be independent of this positive complement regulator. Properdin-dependent and -independent C3NeF types may differ in the degree of convertase stabilization they provide (31) and have been related to different degrees of terminal pathway activation observed in patients (48, 49). A recent study identified properdin-dependent C3NeF as C5NeF: nephritic factors stabilizing C5 convertases (50). Since in our assay whole serum conditions are used in which properdin is present, our assay should be able to detect those factors as well. Furthermore, few previous reports described autoantibodies against the individual components of the convertase, i.e., FB and C3b, which may also increase the convertase half-life (19, 51–53). However, not much is known of the role of these autoantibodies and their occurrence in C3G patients, which tends to be lower than the occurrence of C3NeF (19, 52). In addition, our previous work indicated that samples of aHUS patients positive for FHaAb may also show C3NeF-like stabilizing activity in the assay (37). In this cohort, we also checked for presence of FHaAb, but no patients were found positive. In conclusion, this variety in factors influencing convertase activity *via* different mechanisms may reflect the heterogeneity of the stabilized convertase activity profiles in our cohort. To distinguish between these factors, additional methods using purified components, e.g., enzyme-linked immune sorbent assay (ELISA) techniques, are needed.

In total, 16 out of the 27 patients (59%) of the C3G cohort were positive in this assay (Figure 3B); 67% positive in DDD patients, 57% in the C3GN patients, and 50% in the C3G patient group that could not be subdivided into DDD or C3GN. Of these 16, 13 were children and 3 were adults. Considering that of all convertase-stabilizing factors described earlier C3NeF is most common in C3G, our data are in line with the numbers of C3NeF positive patients reported by other studies (15, 19–21, 54).

Commonly used assays for C3NeF are often based on studying isolated interactions of C3NeF with convertases assembled out of purified proteins on artificial surfaces (ELISA) or on sheep erythrocytes (semiquantitative hemolytic assay). In contrast to RbE, these do not naturally activate the AP and therefore require complex and time-consuming steps to build up the enzyme. Furthermore, in some studies, different detection assays were used alongside each other. Patients can be variably positive in these different assays, again indicating the heterogeneous nature of C3NeF (19, 31).

An important advantage of our method is the direct assessment of convertase stability in whole, unhandled serum, which allows easy screening for any factor directly or indirectly affecting convertase stability, not limited to C3NeF. Moreover, convertases are studied in their physiological surroundings where (negative and positive) complement regulators and other serum factors are present to interact with the convertases. For this reason, we believe our method is particularly suited to detect functionally relevant C3NeF and other stabilizing factors. The assay is also well suited to screen for convertase-influencing factors in other pathologies associated with complement dysregulation, such as age-related macular degeneration, immune complex-mediated membranoproliferative glomerulonephritis, postinfectious glomerulonephritis, systemic lupus erythematosus, and acquired partial lipodystrophy (30, 55, 56). However, future research may focus on further adaptation of the assay with human (renal) cells as platforms for convertase assembly, since they best reflect physiological circumstances regarding the expression of membrane-bound inhibitors and binding places for the soluble inhibitor FH.

A limitation of our assay’s serum approach is that it does not directly give insight into the nature (autoantibody or genetic variant) and mechanism of the convertase-stabilizing factor present in the patient’s serum. When clinically relevant, e.g., to evaluate whether B cell-depleting therapy may be indicated, this nature can be dissected by assessing stabilizing activity in purified Ig fractions, screening for FHaAb, and genetic analysis, as we described in this study.

We have shown that the convertase-stabilizing activity in patients P24–P27 resides in the Ig fraction (Figure 5), indicating the presence of stabilizing autoantibodies. For these patients, we also described the convertase activity during disease progression. In three of them (P24, P27, and P25; Figures 4A–C), convertase-stabilizing factors were present in all samples taken during the different stages of disease. By contrast, the stabilizing Igs detected in the acute phase of P26 became undetectable in remission (Figure 4D). These data are in line with several previous reports on C3G/membranoproliferative glomerulonephritis cohorts that showed a fluctuation in C3NeF activity over disease course in at least one-third of all positive patients (15, 20, 34). Whereas in some patients C3NeF were persistently present, in others they appeared or disappeared during follow-up. Above that, in these studies no correlation was found between C3NeF presence, plasma C3 levels, and clinical recovery. More longitudinal studies are needed to further examine the contribution of C3NeF to disease progression and to establish its value as a (prognostic) disease biomarker.

Even though we did not find patients positive for FHaAb in the described cohort, it is important to screen for these autoantibodies, since they may also indirectly increase convertase stability by impairing the convertase regulation by FH (37). FHaAb occur in around 10% of C3G cases and have different characteristics than those occurring in aHUS cases, for example, regarding their target epitope on FH and their pathological consequences (57, 58). Moreover, FHaAb have been previously reported in simultaneous presence of C3NeF, but the combined role of these two autoantibodies in mediating AP activation remains unclear and requires further investigation (58).

Increased stability of convertases cannot only be caused by autoantibodies but may also be due to genetic aberrations. We confirmed that the stabilizing C3NeF-like activity detected in the aHUS patient with known FB mutation (p.Lys323Glu) is due to its aberrant FB activity: increased convertase stability in serum segregated with the FB mutation in both affected and non-affected family members (Figure 6B), while the purified Igs from this patient did not support convertase stabilization (Figure 6C). This increased convertase half-life associated with the FB mutation is in line with previous functional assays showing that this particular variation causes resistance of the C3 convertase to decay by FH and DAF (41). The fact that a carrier of the mutation shows increased convertase stability in our assay while not being affected with aHUS is supportive for the theory of the incomplete penetrance of mutations in complement genes in aHUS. This theory postulates that multiple (environmental) triggers are required for the actual development of the disease (24). Thus, we show that our assay is also capable as a functional assay for identifying gain-of-function mutations in the building blocks of the AP convertase.

In conclusion, we present optimization of an efficient, robust, and reliable assay for detection and characterization of convertase-stabilizing factors (C3NeF and some genetic changes) in patients with complement-mediated renal diseases and for monitoring these patients during treatment. This assay can be used for studying the role of these factors in the pathophysiology of C3G or related disorders and may contribute to the development of more personalized treatment strategies.

## Ethics Statement

This study was carried out in accordance with the recommendations of the appropriate version of the Declaration of Helsinki. All subjects gave written informed consent, and the protocol was approved by the ethical committee of the Radboud University Medical Center (FH 06979).

## Author Contributions

Concept of the study: NK, MO, AB, EV, and LH. Design of the experiments: MM, MO, EV, and LH. Experimental work: MM and SK. Data analysis: MM, NK, EV, and LH. Collection and characterization of clinical samples (including genetic data): NK, EV, and LH. Manuscript writing: MM. All the authors approved the manuscript.

## Conflict of Interest Statement

NK is a member of the international aHUS Advisory Board of Alexion. The remaining co-authors declare that the research was conducted in the absence of any commercial or financial relationships that could be construed as a potential conflict of interest.

## Abbreviations

aHUS, atypical hemolytic uremic syndrome; AP, alternative pathway; C3G, C3 glomerulopathy; C3GN, C3 glomerulonephritis; C3NeF, C3 nephritic factor; CR1, complement receptor 1; DAF, decay-accelerating factor; DDD, dense deposit disease; EDTA, ethylenediaminetetraacetic acid; EDTA–GVB, EDTA–gelatin veronal buffer; FB, complement factor B; FD, complement factor D; FH, complement factor H; FHaAb, FH autoantibodies; FI, complement factor I; Ig, immunoglobulin; MAC, membrane attack complex; MCP, membrane cofactor protein; Mg–EGTA, magnesium–ethylene glycol tetraacetic acid; NHS, pooled normal human serum; NHP, pooled normal human plasma.
