# Supplementary material for: Overactivity of Alternative Pathway Convertases in Patients With Complement-Mediated Renal Diseases
Source: Front Immunol. 2018 Apr 4;9:612. doi: 10.3389/fimmu.2018.00612 (PMC5893837; doi:10.3389/fimmu.2018.00612)
Supplement: Supplementary file 1 [file data_sheet_1.docx]

Supplementary Material

Overactivity of Alternative Pathway Convertases in Patients with Complement-Mediated Renal Diseases

Marloes A. Michels, Nicole C. van de Kar, Marcin Okrój, Anna M. Blom, Sanne A. van Kraaij, Elena B. Volokhina, Lambertus P. van den Heuvel^*^, on behalf of the COMBAT consortium

*** Correspondence:** [bert.vandenheuvel@radboudumc.nl](mailto:bert.vandenheuvel@radboudumc.nl)

# Supplementary Figures Supplementary Figure 1. Mixing serum 1:1 with pooled normal human serum (NHS) restores convertase activity in samples with low C3 levels. (A) NHS compared to C3-depleted NHS (∆C3-NHS) mixed 1:1 with NHS in the convertase activity assay. Samples were tested with a final serum concentration of 5% and both show effective convertase-mediated hemolysis. ∆C3-NHS was from Complement Technologies. (B) Patient sera tested mixed 1:1 with NHS or unmixed in the convertase activity assay. Patient sera from atypical hemolytic uremic syndrome (aHUS) patient III.6 (PaHUS) and C3 glomerulopathy patient P18 were tested in the two different conditions (mixed or unmixed), both with final serum concentrations of 3.75%. (A and B) Heat-inactivated NHS (∆NHS) and a sample from which guinea pig serum was omitted during the second part of the assay (*no step 2*) served as negative controls for the first and second steps, respectively. Hemolysis levels are presented as percentage of full lysis of erythrocytes in water.

**Supplementary Figure 2.** **Convertase activity profile of serum versus EDTA plasma of a C3 glomerulopathy (C3G) patient.** Both patient samples from P18 were tested mixed 1:1 with pooled normal human serum (NHS) to a final concentration of 5%. Heat-inactivated NHS (∆NHS) and a sample from which guinea pig serum was omitted during the second part of the assay (no step 2) served as negative controls for the first and second steps, respectively. Hemolysis levels are presented as percentage of full lysis of erythrocytes in water.
